# The effect of preoperative degenerative spondylolisthesis on postoperative outcomes of degenerative lumbar spinal stenosis: A single-center cohort study protocol

**DOI:** 10.1097/MD.0000000000022355

**Published:** 2020-11-06

**Authors:** Yueliang Chang, Fubiao Zhou, Le Fei, Zili Wang

**Affiliations:** a Department of Spine Orthopaedic, Ningxia Medical University; b Department of Spine Orthopaedic, General Hospital of Ningxia Medical University, Ningxia, China.

**Keywords:** cohort study protocol, degenerative lumbar spinal stenosis, degenerative lumbar spondylolisthesis, Oswestry Disability Index

## Abstract

**Background:**

Most degenerative lumbar spinal stenosis (DLSS) patients primitively received the conservative treatment to control symptoms. In order to develop an optimal surgical treatment strategy, it is very significant to understand how the degenerative lumbar spondylolisthesis (DS) affects the effect of decompression in the DLSS. Thus, the aim of this current study was to explore whether the concomitant DS would affect the effect of decompression alone in the patients with DLSS.

**Methods:**

The current study was carried out at our hospital and it was approved through our institutional review committee of General Hospital of Ningxia Medical University. During the period from January 2015 to December 2017, in our study, we identified consecutive patients who received the minimally invasive laminectomy to treat the DLSS. The inclusion criterion included radicular leg pain or neurogenic claudication with the neurological symptoms associated with DLSS syndrome, magnetic resonance imaging of the lumbar spine reveals at least 1 level of serious stenosis, the conservative treatment failed for at least 3 months, and patients agreed to provide the postoperative details. The major outcomes of this present research was Oswestry Disability Index. Secondary outcomes of this current study involved visual analog score, short form-36, surgical revision rate as well as complications.

**Results:**

We assumed that previous DS possessed a negative effect on the postoperative results of the DLSS patients.

**Trial registration:**

researchregistry5943.

## 1 Introduction

Degenerative lumbar spinal stenosis (DLSS) is a disease that narrows the intervertebral foramen, lateral recess, or central spinal canal causes the compression of vascular structures and nerve, leading to disability (especially the decline of walking ability), leg and back pain and a significant reduction in health-related life quality.^[1–4]^ Degenerative lumbar spondylolisthesis (DS) is a condition in which a vertebral moves forward relative to the vertebral body below it.^[5–7]^ It affects lower lumbar spine most commonly, which can be seen on the radiographs of some DLSS patients. Some spine surgeons consider this sign of instability to be a mandatory indication of the fusion surgery.^[8]^

Most DLSS patients primitively received the conservative treatment to control symptoms. Nevertheless, surgical treatment is recommended in the case of serious neurological deficits or failure or progression of conservative treatment.^[9–11]^ The minimally invasive laminectomy with similar retractor or tubular is a recently utilized alternative to the DLSS decompression. The key to this technique is to preserve the posterior elements of spine, for instance, intervertebral joints, intraspinal ligaments, supraspinatus ligaments, and paraspinal muscles, and may facilitate the preservation of stable spinal ligaments and the bone structures.^[12–16]^ Because the preservation of the posterior component can minimize the damage to instability or scoliosis after the decompression, indications are extended to the lumbar diseases, involving DS or DLSS. Nevertheless, there is no consensus on these indications.^[17–20]^

In order to develop an optimal surgical treatment strategy, it is very significant to understand how the DS affects the effect of decompression in the DLSS. If there is no difference between DS patients and non-DS patients after the simple decompression surgery, it is doubtful whether some patients will need to undergo fusion. Thus, the aim of this current study was to explore whether the concomitant DS would affect the effect of decompression alone in the patients with DLSS. We assumed that previous DS possessed a negative effect on the postoperative results of the DLSS patients.

## 2 Materials and methods

### 2.1 Trial design

The current study was carried out at our hospital and it was approved through our institutional review committee of General Hospital of Ningxia Medical University (DX2020-07-31). During the period from January, 2015 to December, 2017, in our study, we identified consecutive patients who received the minimally invasive laminectomy to treat the DLSS. The data for this retrospective case series were obtained from the hospital database collected prospectively. All of trial surgeons were senior consultants with extensive experience in the implementation of both trial interventions. This study scheme has been registered with research registry (researchregistry5943).

### 2.2 Patient population

The inclusion criterion included radicular leg pain or neurogenic claudication with the neurological symptoms associated with DLSS syndrome, magnetic resonance imaging of the lumbar spine reveals at least 1 level of serious stenosis, the conservative treatment failed for at least 3 months, and patients agreed to provide the postoperative details. Patients were excluded for these reasons: with the history of lumbar spinal surgery for the instability or lumbar stenosis, the degenerative lumbar scoliosis (the angle of Cobb greater than 20 degrees), stenosis due to the disc herniation, neurological disease, cancer, and ankylosing spondylitis, or the history of the vertebral compression fractures of the affected segments.

### 2.3 Operative techniques

After the patient was placed in prone position, conducting the midline skin incision, and then unilateral access to the relevant interlaminar space. Laminectomy was carried out at the insertion site of ligamentum flavum, and the articular process resection was conducted with the trumpeted means until the medial side of pedicle, and the microscope was slightly tilted laterally. The basal part of the spinous process of the caudal half of the cranial lamina and a small cranial portion of the caudal lamina were removed through utilizing a high-speed drill. After removing yellow ligament, the decompression of contralateral nerve root was conducted; and the effectiveness of decompression was confirmed by observing the process on the medial side of the pedicle.conducted; and the effectiveness of decompression was demonstrated through observing the process on the medial side of pedicle.

### 2.4 Data collection

Part of the baseline data was interviewed, managed and then recorded via the research coordinator. The other questionnaires were self-filled questionnaires and filled via patients themselves. All the data were harvested during baseline and after 1, 2, and 3 years. The research coordinator checked the integrity of all the questionnaires. In the case of data loss, he would call the patient and attempt to retrieve the lost data. The data was independently input, repeated in 2 databases and cross checked. Any discrepancies in the primary document were reviewed.

### 2.5 Outcome measures

The major outcomes of this present research was Oswestry Disability Index. Oswestry Disability Index includes 10 items about the severity of leg or back diseases that influence the ability to manage daily living. These 10 components include the daily functions and pain (containing personal hygiene, pain intensity, sitting, walking, lifting, sleeping, standing, and traveling, sexual activity, as well as social activity). Each item will be scored on a 6-point scale (0–5); the higher the score, the higher the degree of disability associated with the lower back pain.

Secondary outcomes of this current study involved visual analog score, short form-36 (SF-36), surgical revision rate as well as complications. The SF-36 determines 8 indicators: physical pain, role physiology, physical function, social function, vitality, and general health, mental health, and role emotional. We chose the SF-36 average bodily pain score and average physical score to perform the analysis (Table 1).

**Table 1:** Postoperative outcomes.

Outcomes	Group A	Group B	*P* value
ODI			
Short form-36			
Visual analog score			
Revision rate			
Complications			

### 2.6 Statistical analysis

The paired t-test, Mann–Whitney *U* test, and Chi-squared test, the Spearman rank correlation coefficient, as well as the Pearson product-moment correlation coefficient were utilized for the statistical analyses. The comparison of demographic characteristics between the two groups was carried out through utilizing the descriptive statistics method. All the analyses were implemented with the software of StatView-J 5.0 (SAS Institute, Inc.). The value of *P* < .05 was considered as significant.

## 3 Discussion

DLSS is a familiar degenerative spondylotic disease in the elderly. The symptoms of DLSS involve leg and back pain along with claudication. In DLSS cases, the leg and back pain associated with intermittent claudication are caused by ischemia and compression of nerve root or cauda equina. DS is a condition in which a vertebral moves forward relative to the vertebral body below it, which can be seen on the radiographs of some DLSS patients. Whether the DS is present or not, there is insufficient evidence that more complex decompression combined with the fusion is more advantageous than the decompression alone. In addition, the long-term clinical advantages of the microendoscopic laminotomy for parents with LSS combined with DS are unclear. Thus, the aim of this current study was to explore whether the concomitant DS would affect the effect of individual decompression in the patients with DLSS. We assumed that previous DS possessed a negative effect on the postoperative results of the DLSS patients. Our research required the retrospective data collection and the analysis, which may lead to patient selection confusion and bias.

## Author contributions

**Conceptualization:** Yueliang Chang, Zili Wang.

**Data curation:** Yueliang Chang, Fubiao Zhou, Le Fei

**Formal analysis:** Yueliang Chang, Fubiao Zhou, Le Fei.

**Funding acquisition:** Zili Wang.

**Investigation:** Yueliang Chang, Fubiao Zhou, Le Fei.

**Methodology:** Zili Wang.

**Project administration:** Zili Wang.

**Resources:** Zili Wang.

**Software:** Le Fei

**Supervision:** Zili Wang.

**Validation:** Fubiao Zhou

**Validation:** Le Fei.

**Visualization:** Le Fei, Fubiao Zhou.

**Writing – original draft:** Yueliang Chang.

**Writing – review & editing:** Zili Wang.
